# Post-surgical scar management and rehabilitation in burn patients: Insights from Gaza’s challenging context - A retrospective descriptive study

**DOI:** 10.1371/journal.pgph.0006168

**Published:** 2026-07-07

**Authors:** Abed El Hamid Qaradaya, Julie Van Hulse, Jomaa Younis, Feras Swairjo, Hala Al Far, Noura Al Zaeem, Ebtihal Wally, Mohamad Abu Hashem, Rami Al Shamali, Mabade Al Farra, Natalie Thurtle, Mohammed Abu Mughiaseeb, Mohamad Al Ghazali, Jihane Ben Farhat

**Affiliations:** 1 Médecins Sans Frontières, Paris, France; 2 Epicentre, Médecins Sans Frontières, Department of Epidemiology and Training, Paris, France; Universidad Nacional de Colombia, COLOMBIA

## Abstract

Burn injuries represent a challenge in the Gaza Strip, where access to rehabilitation services is constrained by ongoing conflict and limited healthcare resources. This study describes the implementation and outcomes of rehabilitation and physiotherapy services for patients with post-surgical skin grafts (SSG) scars treated in Médecins Sans Frontières (MSF) outpatient clinics in Gaza. We conducted a retrospective descriptive study using routinely collected programmatic data from patients with SSG scars enrolled in MSF post-operative outpatient clinics in the Gaza Strip (Gaza City, Beit Lahia, and Khan Younis), between January 2018 and December 2020. Outcomes were assessed using five measures: reducibility score, Functional Activity for Burn (FAB) score, Vancouver Scar Scale (VSS), Visual Analogue Scale (VAS) for pain, and an itching score. A total of 177 patient records were included. Most patients were children under 18 years (n = 136, 76.8%), and scald burns were the most common injury mechanism (n = 119, 67.6%). Improvements were observed across several clinical and patient-reported outcomes during follow-up. Mean pain scores decreased from 5.3 (SD: 2.5) to 1.4 (SD: 1.8), itching scores from 3.7 (SD: 2.7) to 2.7 (SD: 2.2), and VSS scores from 7.1 (SD: 1.8) to 5.7 (SD: 1.7). Functional outcomes improved, with mean FAB scores increasing from 25.6 (SD: 7.1) to 34.6 (SD: 2.3), while reducibility scores decreased from 2.3 (SD: 1.4) to 0.8 (SD: 0.9). Our findings describe the implementation of rehabilitation and physiotherapy services and document improvements observed during follow-up among burn patients in the Gaza Strip. Despite the challenging environment, MSF’s clinic in Gaza demonstrated the feasibility of delivering rehabilitation care to burn survivors in a conflict-affected area. Further research is needed to refine and validate best practices in rehabilitation interventions tailored to the specific needs of patients in conflict-affected and resource-constrained settings and to continue improving the quality of care for burn survivors.

## Background

The Gaza Strip, nestled amidst ongoing socio-political strife, stands as an emblem of the enduring repercussions of conflict. Characterized by its constrained geographical expanse and a staggering population density of 5,046 inhabitants per km2, this territory grapples with multifaceted challenges exacerbated by socio-political strife. The blockade imposed by Israel has further exacerbated the plight of its inhabitants, with over a million people – nearly half the population – pushed below the poverty line in just over a decade [1,2]. Within this backdrop, injuries, both domestic and conflict-related, impose a heavy toll, underscoring the critical need for robust and adaptive care strategies. In Gaza, the aftermath of burns transcends mere physical trauma, often culminating in hypertrophic scars, contractures, and long-term functional limitations. Although several scar management interventions have been described, evidence regarding their implementation and outcomes in pediatric populations and resource-constrained settings remains limited [3,4]. Amidst socio-political challenges, Médecins Sans Frontières (MSF) has been providing vital care, including specialized physiotherapy and rehabilitation services.

Hypertrophic scars result from an overabundance of fibroblast-produced extracellular matrix proteins, notably collagen, persisting over extended durations. These scars pose significant risks, including skin contractures and impairments in both range of motion and functional capacity. Skin graft procedures similarly predispose individuals to hypertrophic scarring, exacerbating these enduring complications. Numerous therapeutic approaches have been devised to address post-burn scarring, each subjected to rigorous evaluation through research endeavors. A systematic review of diverse studies indicates that employing pressure garments for a minimum of 23 hours daily over 6–12 months, with pressures ranging from 20 to 40 mmHg, can effectively enhance scar mobility in joint regions, forestall contractures, and mitigate the risk of scar contraction [4]. Additionally, the utilization of silicone sheets and gels is advocated for both the prevention and treatment of hypertrophic scars and keloids. Research underscores the significant benefits of topical silicone sheets, manifesting in notable improvements in the color, thickness, and suppleness of hypertrophic scars and keloids [3].

Few studies have explored scar management techniques, particularly for post-surgical scars in pediatric populations. Treating burns in children presents unique challenges owing to their developmental stage and rapid growth rate. Scar treatment in this demographic necessitates a comprehensive rehabilitation regimen, encompassing diverse modalities such as scar tissue manipulation, joint mobilization, functional rehabilitation to facilitate daily activities, and utilization of various compression tools and materials. This holistic approach is pivotal in curbing scar progression over the protracted maturation period, which can extend up to two years.

Médecins Sans Frontières (MSF) started working in the Occupied Palestinian Territories in 1989, to treat victims of the Israeli-Palestinian conflict. In 2000, MSF began activity in Gaza with a psychological project and in 2007, then launched a medical and rehabilitation project to treat victims of trauma and burns. For the last 14 years the project has treated multiple forms of trauma including injuries linked to violence, domestic burns injuries, and hand injuries. A physiotherapy and rehabilitation department forms part of the programme, providing rehabilitation through a multidisciplinary team. In 2020, 11,330 physiotherapy sessions were provided to burns patients, with an average of 14 sessions per patient over the course of treatment. In the same year, 89 burn patients received skin graft surgery from the MSF clinic. Most patients in this project are children who have experienced domestic burns accidents. MSF has a specialized physiotherapy and rehabilitation protocol for treating burns patients; the protocol focuses on providing scar tissue massage (using gradual divergent, multi-axis two-handed massage, detaching and folding the skin, and skin release and torsion), skin stretching techniques, functional rehabilitation and mobility exercises, scar tissue hydration using cold creams, and compression therapy involving pressure garments, cohesive bandages, and tubular elastic bandage. Finally, insert materials are also used such as silicone Sheets, soft putty elastomer, and polyethylene foam (Plastazote).

We aimed to describe the patients who received rehabilitation/physiotherapy care for post-surgical skin grafts. We also aimed to describe the use of materials like silicone and soft putty for scar management and outline the characteristics and outcomes of these patients.

## Methods

### Ethics statement

This study used retrospectively collected routine programmatic data from Médecins Sans Frontières (MSF) clinical services in Gaza. The study protocol was reviewed and approved by the Palestinian Health Research Council (PHRC) – Helsinki Committee (Approval number: PHRC/HC/907/21).

As the study involved analysis of anonymized routine clinical data, a waiver of formal ethics review was granted by the MSF Operational Centre Paris Medical Directorate, in accordance with MSF procedures for retrospective analyses. The study was conducted with the knowledge and approval of the Gaza Strip Ministry of Health. During their first clinical visit, patients provide written consent allowing the use of their anonymized clinical data for research purposes. All data were anonymized prior to analysis.

### Study site

The study took place at the burn center, which had been established in 2007 and is located in Nasser Medical Complex in the Gaza Strip. It serves more than 1.1 million inhabitants and covers all Gaza governorates except the southern governorates. It has 12 beds distributed in 6 rooms in addition to a dressing and hydrotherapy room. Furthermore, three ICU beds and one operating room are available in service. Care is provided by plastic surgeons, nurses and physiotherapists, antimicrobial stewardship doctors, as well as psychological support from psychologists.

### Design

This study employed a retrospective descriptive design, involving patients who underwent skin graft surgery as part of MSF’s physiotherapy and rehabilitation program in the Gaza Strip from January 1st, 2018, to December 31st, 2020. Eligible patients had post-surgical skin graft (SSG) scars resulting from burns during the study period and were discharged from clinic. To assess clinical and functional outcomes following physiotherapy, five outcome measures routinely used in burn rehabilitation were collected at admission and discharge. Scar contracture and tissue flexibility were assessed using the Reducibility Score, an operational assessment tool described in the MSF OCP Burns Guidelines, ranging from 0 (best reducibility) to 4 (worst reducibility) [5]. Functional recovery was evaluated using the Functional Assessment for Burns (FAB) score, a burn-specific functional outcome measure ranging from 7 (worst function) to 35 (best function) [6]. Scar characteristics were assessed using the validated Vancouver Scar Scale (VSS), which evaluates pigmentation, vascularity, pliability, and scar height, with scores ranging from 0 (best outcome) to 13 (worst outcome) [7]. Pain intensity was measured using the validated Visual Analogue Scale (VAS), ranging from 0 (no pain) to 10 (worst imaginable pain) [8]. Finally, pruritus severity was assessed using a numerical rating itching scale ranging from 0 (no itching) to 10 (severe itching), a commonly used approach for itch assessment [9].

### Data collection

Data were accessed for research purposes between August and October 2021. All data were extracted from routine clinical records and anonymized prior to analysis. The authors did not have access to information that could identify individual participants during or after data collection. Data were entered into RedCap (Vanderbilt University). The demographic information gathered for each patient encompassed age, sex, employment, and educational status, as well as any post-burn related comorbidities. Additionally, details regarding injury characteristics, including cause, location, type, pain levels, reducibility scores, scar assessments, and surgical interventions such as operations and procedures, along with their timing, were documented. Furthermore, information on the specific physiotherapy and rehabilitation modalities employed for each patient was recorded, encompassing the treatment scheme, utilized materials (such as silicone sheets, soft putty elastomer, and polyethylene foam), pain management strategies, itching management, treatment frequency and timing, as well as injury and episode outcomes.

### Statistical analysis

We analyzed the data descriptively, providing details on the median and inter-quartile range (IQR) for numeric variables and proportions for categorical variables. The total number of non-missing values served as the denominator for calculations. Additionally, differences between outcomes before and after treatment were calculated, including mean difference and standard deviation. Data was analyzed using Stata V.15 software (Stata Corporation, Texas, USA).

## Results

### Demographics and subject characteristics

A total of 240 patient records meeting the programme eligibility criteria were identified. These included patients who underwent skin graft surgery and completed physiotherapy treatment between January 1st, 2018, and December 31st, 2020. Of these, 63 records lacked sufficient clinical outcome information for analysis and were therefore excluded. The final analytic sample consisted of 177 patients (73.8%) (Table 1). The study population was young, with a median age of 5 years (IQR 2–13), and the majority were under 18 years old (n = 136; 76.8%). Males accounted for 56.5% of the population (n = 100). The prevalence of post-burn related comorbidities was low, with only one patient having undergone a fasciotomy. Over 70% of the study population sustained minor burns (children: n = 95, 73.1%; adults: n = 28, 70%), predominantly caused by scalds (n = 119, 67.2%) (Fig A in S1 Appendix). Most injuries affected the upper limbs (n = 106, 56.9%), with corresponding graft sites primarily located on the upper limbs (n = 91, 52.4%) (Table 2). The thigh area was the most common donor skin site (n = 167, 49.3%) (Table 2).

**Table 1:** Characteristics of study population.

Characteristics	N = 177	
Age, median [IQR], years	5	[2-13]
Age groups in years, n (%)		
<1	29	(16.4)
1-5	63	(35.6)
6-10	31	(17.5)
11-19	15	(8.5)
≥20	39	(22.0)
Child vs Adult, n (%)		
Child 0–18 y.o.	136	(76.8)
Adult 19+	41	(23.2)
Gender, n (%)		
Female	77	(43.5)
Male	100	(56.5)
Comorbidities, n (%)		
Fasciotomy	1	(0.6)
None	176	(99.4)

*IQR = Interquartile Range.

**Table 2:** Location of burns and sites of graft.

	Location of burns n (%)	Sites of grafts n (%)	Donor site	n (%)
Left Upper limb	50 (25.3)	46 (26.0)	Left thigh	79 (44.6)
Right upper limb	56 (31.6)	45 (25.4)	Right thigh	88 (49.7)
Left hand	24 (13.6)	17 (9.6)	Left leg	–
Right hand	28 (15.8)	25 (14.1)	Right leg	3 (1.7)
Left lower limb	42 (23.7)	35 (19.8)	Trunk	6 (3.4)
Right lower limb	42 (23.7)	35 (19.8)	Other	16 (9.0)
Anterior trunk	33 (18.6)	23 (13.0)		
Posterior trunk	14 (7.9)	7 (4.0)		
Face and head	14 (7.9)	6 (3.4)		

*Patients could present with more than one injury type; therefore percentages do not sum to 100%.

The mean delay between surgery and admission for physiotherapy was 13.2 days, while the average duration of physiotherapy treatment was 178.9 days, equivalent to approximately 5.9 months.

### Primary outcomes

Fig B in S1 Appendix shows physiotherapists predominantly relied on tube grip (77 patients, 44.5%) and pressure garments (61 patients, 35.3%) as primary methods for pressure therapy treatment. Among these, a majority of patients (40 patients, 82.4%) received pressure therapy alone, without any additional insert material (Fig B in S1 Appendix). Conversely, 146 patients underwent pressure therapy in conjunction with insert materials. Silicone sheets emerged as the most frequently utilized insert material (92 patients, 63%), followed by polyethylene foam plastazote (45 patients, 30.8%), with only 9 patients receiving soft putty (6.1%) (Refer to Table 3). On average, insert materials were utilized for a duration of 119.8 days (SD = 82.5).

**Table 3:** Mean change in outcome scores between admission and discharge according to insert material used.

	Total (n)	Average Point Gained in pain score (initial score - final score)	Average Point Gained in Reducibility score (initial score - final score)	Average Point Gained in FAB score (initial score - final score)	Average Point Gained in Scar assessment VSS (initial score - final score)	Average Point Gained in Itching Score (initial score - final score)
Silicone sheet	92	4.11	1.61	4.88	1.60	0.78
Soft putty elastomer	9	3.56	1.67	7.00	0.78	1.89
Polyethylene foam (Plastazote)	45	4.16	1.40	5.34	1.47	0.93

Note. Values represent the mean change between admission and discharge scores among patients who received each insert material. For pain, reducibility, Vancouver Scar Scale (VSS), and itching scores, positive values indicate a reduction in symptom severity or scar burden. For the Functional Assessment for Burns (FAB) score, positive values indicate improved functional ability. Patients could receive more than one pressure therapy modality during treatment.

The majority of patients attended on at least 3 physiotherapy sessions per week (131, 74.0%) while fewer patients (39, 22%) attended 2 sessions per week. Most patients were discharged as cured (153, 86.4%), however 23 patients were discharged as defaulters and were lost to follow-up (13.0%), and one patient was transferred to another facility (0.6%).

The findings in Tables D-H in S1 Appendix indicate that post-surgical skin graft physiotherapy led to improvements across various scar characteristics. The Vancouver Scar Scale (VSS) demonstrated a reduction in scar pigmentations, vascularity, and height, with enrolled patients showing a decrease from a mean initial VSS of 7.1 (SD, 1.8) to a mean final of 5.7 (SD, 1.7). Additionally, there was a notable decrease in pain levels, as reflected by the Visual Analogue Scale (VAS) scores, declining from a mean initial VAS of 5.3 (SD, 2.5) to a mean final score of 1.4 (SD, 1.8). Furthermore, patients experienced a reduction in scar itching, with the mean initial itching score decreasing from 3.7 (SD, 2.7) to a mean final score of 2.7 (SD, 2.2). Scar contracture and reducibility also improved, as evidenced by a decline in the Reducibility score from a mean initial score of 2.3 (SD, 1.4) to a mean final score of 0.8 (SD, 0.9). Moreover, there was a notable enhancement in functional abilities among enrolled patients, as measured by the Functional Activity for Burn (FAB) score, which increased from a mean initial score of 25.6 (SD, 7.1) to a mean final score of 34.6 (SD, 2.3).

### Secondary outcomes

The provided data in Table 3 illustrate the outcomes associated with different insert materials used in pressure therapy, along with the corresponding total number of patients. Among the 92 patients treated with silicone sheets, there was an average gain of 4.11 points in pain score, 1.61 points in Reducibility score, 4.88 points in FAB score, 1.60 points in scar assessment VSS, and 0.78 points in itching score. For the 9 patients treated with soft putty, the average gains were 3.56 points in pain score, 1.67 points in Reducibility score, 7.00 points in FAB score, 0.78 points in scar assessment VSS, and 1.89 points in itching score. Among the 45 patients treated with polyethylene foam plastazote, there was an average gain of 4.16 points in pain score, 1.40 points in Reducibility score, 5.34 points in FAB score, 1.47 points in scar assessment VSS, and 0.93 points in itching score.

## Discussion

Our study describes improvements in patient-reported and clinical outcomes observed during follow-up among patients receiving comprehensive physiotherapy treatment amidst the challenging backdrop of conflict and resource limitations in Gaza. By focusing on scar hypertrophy management following skin graft surgery, our findings shed light on the implementation and observed outcomes of a tailored rehabilitation program adapted to the unique needs of burn patients, particularly children. One noteworthy aspect of our findings is the short interval observed between surgery and physiotherapy initiation, with patients starting rehabilitation approximately two weeks after surgery on average. This highlights the importance of timely access to rehabilitation services, especially in resource-constrained environments and conflict contexts. Furthermore, our observation that silicone sheets were the predominant material used in our program highlights the practical considerations inherent in delivering scar management interventions in conflict settings. Despite the challenges posed by the blockade and limited access to medical supplies, our program has implemented rehabilitation practices adapted to the local context. Importantly, our study contributes novel insights to the literature on interventions and programs in conflict-affected and resource-limited settings. By describing the implementation of comprehensive physiotherapy support in Gaza and documenting patient outcomes over time, we provide useful operational insights for similar initiatives grappling with the dual challenges of conflict and constrained resources. Moving forward, it is imperative to leverage our findings to advocate for the expansion and sustainability of such programs in Gaza and beyond. By advocating for increased investment in rehabilitation services within conflict settings, we can ensure equitable access to essential care for those most vulnerable to the enduring repercussions of conflict-related injuries.

The demographic characteristics of our study cohort closely resembled those outlined in a prior descriptive study conducted at the Al Alamy burn center, the primary healthcare facility in the Gaza Strip. This study, published in 2016, offered insights into the epidemiological profile and outcomes among hospitalized burn patients [10]. In line with published literature, our study underscores the predominance of pediatric burn injuries, with the upper body emerging as the most commonly affected anatomical region. This pattern aligns with existing data attributing a significant portion of pediatric burn injuries to scalds resulting from contact with hot liquids [10]. The choice of the thigh as the primary donor site for skin grafts, as observed in our study, reflects established surgical practices aimed at minimizing donor site morbidity and optimizing graft survival [11]. This underscores the importance of surgical precision and careful consideration of grafting techniques in achieving favorable outcomes in burn patients.

The timing and initiation of post-surgical physiotherapy are considered important components of burn rehabilitation programmes [5]. Our study reveals that physiotherapy initiation, occurring approximately two weeks post-surgery on average, aligns with rehabilitation protocols designed to support wound healing and recovery [5]. Notably, the protracted duration of rehabilitation, averaging six months in our cohort, underscores the prolonged nature of scar maturation and the importance of sustained therapeutic interventions during this critical period [12]. This highlights the need for comprehensive, multidisciplinary rehabilitation programs tailored to the unique needs of burn patients to optimize functional outcomes and quality of life.

Effective scar management is paramount in burn rehabilitation, with pressure therapy and insert materials playing pivotal roles in optimizing scar outcomes [4]. Our findings show that tube grip and pressure garments were the most commonly used pressure therapy modalities within the programme. While our study was not designed to assess effectiveness, these findings provide a description of scar management practices in this setting. However, considerations of cost-effectiveness and resource availability underscore the need for context-specific approaches, particularly in resource-limited settings where alternative materials such as polyethylene foam and soft putty elastomer may offer viable alternatives [13]. This highlights the importance of tailoring scar management strategies to individual patient needs and local resource availability.

Patient adherence and engagement emerge as central themes in our study, with high rates of treatment compliance and post-treatment follow-up suggesting good engagement with rehabilitation services despite the challenges associated with care delivery in this context. Despite socio-economic adversities and logistical challenges, our findings underscore the importance of patient education, psychosocial support, and community-based interventions in fostering continuity of care and optimizing treatment outcomes. This underscores the need for holistic, patient-centered approaches to burn rehabilitation that prioritize patient empowerment and engagement throughout the treatment continuum.

Pressure therapy has emerged as a cornerstone in the management of hypertrophic scars following burn injuries [4]. Pressure garments are widely used in burn rehabilitation and are recommended in the literature for scar management. Their frequent use in our programme is consistent with these recommendations [4]. The predominant use of pressure garments in our study population reflects the pragmatic adaptation of standard protocols to resource-constrained settings, illustrating how scar management interventions can be implemented in conflict-affected regions such as the Gaza Strip. Furthermore, our observations regarding recommended pressure levels align with empirical evidence, emphasizing the importance of balancing scar maturation with patient comfort and tolerance [4]. However, further research is warranted to refine recommendations regarding pressure duration and intensity, considering the variability in scar response and patient characteristics [4].

Silicone therapy represents another cornerstone in scar management, supported by extensive clinical literature describing its use in the prevention and management of hypertrophic scars and keloids [3]. Silicone therapy was commonly used within our programme, reflecting its incorporation into contemporary scar management protocols, particularly in early scar maturation stages [3]. The simplicity, non-invasiveness, and favorable side effect profile of silicone products make them invaluable adjuncts to pressure therapy, offering additional benefits in scar pliability, vascularity, and aesthetics [14]. However, ongoing research is needed to elucidate the mechanisms underlying silicone therapy’s efficacy and to optimize treatment protocols, particularly in resource-constrained settings where access to specialized care may be limited [3].

Emerging materials such as thermoplastic polyethylene foam and soft putty elastomer offer promising alternatives to traditional silicone therapy, albeit with limited clinical data [13]. Our study underscores the importance of exploring novel materials and technologies to enhance scar management outcomes, particularly in settings where access to specialized resources may be limited [13]. Future research endeavors should prioritize rigorous clinical trials to validate the efficacy and safety of these emerging materials, considering factors such as patient comfort, durability, and cost-effectiveness. Additionally, interdisciplinary collaboration and knowledge sharing are essential to drive innovation in scar management and improve outcomes for patients worldwide.

While our study provides valuable insights into the implementation and observed outcomes of pressure therapy and silicone therapy in scar management, several challenges and knowledge gaps remain. The socio-political context of the Gaza Strip presents unique challenges to healthcare delivery, including limited access to specialized resources, infrastructure constraints, and political instability [2]. Addressing these challenges requires a multifaceted approach, encompassing advocacy, capacity building, and international collaboration to enhance the resilience and sustainability of burn care services in conflict-affected regions [2]. Furthermore, future research endeavors should prioritize patient-centered outcomes, community engagement, and implementation science to ensure the effective translation of research findings into clinical practice.

The limitations of our study are rooted in its retrospective design and absence of a control group. This inherent design constraint hampers our ability to establish causal relationships between interventions and observed outcomes. Consequently, while our findings illuminate trends in treatment outcomes over time, caution must be taken in attributing these improvements solely to the interventions administered. Moreover, the absence of a control group precludes definitive comparisons between different interventions. Although our study provides valuable insights into the utilization and outcomes associated with pressure therapy methods and insert materials, the lack of a comparative arm limits the robustness of our conclusions. In addition, 63 records meeting programme eligibility criteria were excluded because key clinical outcome data were unavailable. As limited information was available for these records, we were unable to compare included and excluded patients, and the possibility of selection bias cannot be excluded. Additionally, the small sample size further underscores the need for cautious interpretation of our findings. With only nine patients receiving soft putty elastomer inserts compared to 92 patients receiving silicone sheets, the uneven distribution of interventions restricts the generalizability of our results. Furthermore, the coexistence of concurrent pressure therapy methods and variability in clinician-driven treatment selection may introduce confounding factors, complicating the interpretation of outcomes. While our study sheds light on the practicalities and outcomes of scar management interventions in a conflict-affected setting, the retrospective design, absence of a control group, and small sample size collectively temper the strength of our conclusions and highlight avenues for future research and improvement. In addition, although written consent for the use of anonymized data was obtained at the first clinical visit, this occurred in a context of acute care and vulnerability. Patients and caregivers may have had limited capacity to fully consider the implications of data use for research purposes, which represents an inherent limitation of research conducted in humanitarian settings. Further research using prospective methods, a prespecified sample size and sufficient power to compare outcomes between treatment groups, would help inform further on which methods, materials, and inserts best support recovery for burn patients in such contexts.

## Conclusion

In conclusion, our study describes the implementation of post-surgical physiotherapy and pressure therapy with insert materials and documents improvements observed over the course of rehabilitation among patients recovering from burn scars in a conflict-affected setting. Focused on the post-surgical phase following skin graft procedures, our findings document changes observed during rehabilitation in scar characteristics, pain, itching, contracture, and functional outcomes. Given the impact that burn scars and functional limitations can have on daily life, such improvements may have implications for overall wellbeing, although this was not directly evaluated. The integration of physiotherapy and pressure therapy into comprehensive rehabilitation programs may represent a useful approach for supporting scar management and functional recovery in burn patients. While our study provides valuable insights into the use of these interventions in routine clinical practice, further research is needed to optimize treatment protocols and refine recommendations for clinical practice. Future studies should focus on elucidating the mechanisms underlying the therapeutic effects of physiotherapy and pressure therapy, as well as evaluating long-term outcomes and cost-effectiveness.

## Supporting information

S1 Appendix Table A: Total surface body area, by age group (child 0–18, Adult 19+). Table B: Delay between injury, surgery, and admission. Table C: Days between admission to physiotherapy and discharge. Table D: Scar assessment initial and final (VSS); n(%). Table E: Pain Scores from initial to final (VAS); n (%). Table F: Itching Scores, initial and final; n (%). Table G: Contracture Scores initial and final (Reducibility Score); n (%). Table H: Functional Ability Scores initial and final (FAB); n (%). Fig A: Cause of burn. Fig B: Pressure therapy methods. (DOCX)

## References

[1] Palestinian Central Bureau of Statistics. Annual Palestinian Statistics books [Internet]. [cited 2026 Jun 2]. Available from: http://www.pcbs.gov.ps

[2] United Nations. UN report finds Gaza suffered $16.7 billion loss from siege and occupation | UN News [Internet]. [cited 2026 Jun 2]. Available from: https://news.un.org/en/story/2020/11/1078532

[3] Meaume S, Le Pillouer-Prost A, Richert B, Roseeuw D, Vadoud J. Management of scars: updated practical guidelines and use of silicones. Eur J Dermatol. 2014;24(4):435–43. doi: 10.1684/ejd.2014.2356 25141160

[4] Ai J-W, Liu J-T, Pei S-D, Liu Y, Li D-S, Lin H-M, et al. The effectiveness of pressure therapy (15-25 mmHg) for hypertrophic burn scars: A systematic review and meta-analysis. Sci Rep. 2017;7:40185. doi: 10.1038/srep40185 28054644 PMC5215680

[5] Ocp MSF. GUIDELINES. 2019;(January).

[6] Smailes ST, Engelsman K, Dziewulski P. Physical functional outcome assessment of patients with major burns admitted to a UK Burn Intensive Care Unit. Burns. 2013;39(1):37–43. doi: 10.1016/j.burns.2012.05.007 22677162

[7] Baryza MJ, Baryza GA. The Vancouver Scar Scale: an administration tool and its interrater reliability. J Burn Care Rehabil. 1995;16(5):535–8. doi: 10.1097/00004630-199509000-00013 8537427

[8] Cline ME, Herman J, Shaw ER, Morton RD. Standardization of the visual analogue scale. Nurs Res. 1992;41(6):378–80. doi: 10.1097/00006199-199211000-00013 1437591

[9] Pereira MP, Ständer S. Assessment of severity and burden of pruritus. Allergol Int. 2017;66(1):3–7. doi: 10.1016/j.alit.2016.08.009 27634668

[10] Elsous A, Ouda M, Mohsen S, Al-Shaikh M, Mokayad S, Abo-Shaban N, et al. Epidemiology and Outcomes of Hospitalized Burn Patients in Gaza Strip: A Descriptive Study. Ethiop J Health Sci. 2016;26(1):9–16. doi: 10.4314/ejhs.v26i1.4 26949311 PMC4762954

[11] Hariharan N, Sridhar R, Sankari B, Valarmathy V, Asirvatham E, Geetha K. Reconstruction of postburn crippled hands: A study of functional outcome. Indian J Burns. 2018;26(1):9. doi: 10.4103/ijb.ijb_20_18

[12] Kant S, van den Kerckhove E, Colla C, van der Hulst R, Piatkowski de Grzymala A. Duration of scar maturation: retrospective analyses of 361 hypertrophic scars over 5 years. Adv Skin Wound Care. 2019;32(1):26–34. 30531549 10.1097/01.ASW.0000547415.38888.c4

[13] Yu A, Yick KL, Ng SP, Yip J. Orthopaedic textile inserts for pressure treatment of hypertrophic scars. Text Res J. 2016;86(14):1549–62.

[14] Berman B, Perez OA, Konda S, Kohut BE, Viera MH, Delgado S, et al. A review of the biologic effects, clinical efficacy, and safety of silicone elastomer sheeting for hypertrophic and keloid scar treatment and management. Dermatol Surg Off Publ Am Soc Dermatologic Surg. 2007;33(11):1291–302; discussion 1302-3. doi: 10.1111/j.1524-4725.2007.33280.x 17958580

