# Supplementary material for: Post-surgical scar management and rehabilitation in burn patients: Insights from Gaza’s challenging context - A retrospective descriptive study
Source: PLOS Glob Public Health. 2026 Jul 7;6(7):e0006168. doi: 10.1371/journal.pgph.0006168 (PMC13340852; doi:10.1371/journal.pgph.0006168)
Supplement: S1 Appendix — (DOCX) [file pgph.0006168.s001.docx]

**Supplementary materials**

Table A: Total surface body area, by age group (child 0-18, Adult 19+)

Table B: Delay between injury, surgery, and admission

Table C: Days between admission to physiotherapy and discharge

Table D: Scar assessment initial and final (VSS); n(%)

Table E: Pain Scores from initial to final (VAS); n (%)

Table F: Itching Scores, initial and final; n (%)

Table G: Contracture Scores initial and final (Reducibility Score); n (%)

Table H: Functional Ability Scores initial and final (FAB); n (%)

Fig A: Cause of burn

Fig B: Pressure therapy methods

Table A: Total surface body area, by age group (child 0-18, Adult 19+)

|  | Children  n (%) | Adults  n (%) |
| --- | --- | --- |
| Minor | 95 (73.1) | 28 (70.0) |
| Moderate | 21 (16.2) | 7 (17.5) |
| Major | 14 (10.8) | 5 (12.5) |
| *Missing* | 6 | 1 |


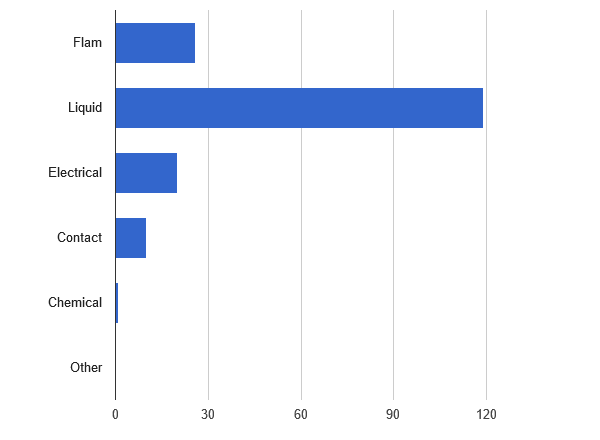


Fig A: Cause of burn

Table B: Delay between injury, surgery, and admission

|  | **Average** | **SD** |
| --- | --- | --- |
| Average delay between surgery to admission | 13.2 | 75.1 |
| Average delay between admission to discharge | 178.9 | 109.3 |
| Average delay between surgery to discharge | 165.7 | 91.6 |

Fig B: pressure therapy methods

Table C: Days between admission to physiotherapy and discharge

|  | **Number enrolled** | **Mean days in care** | **p25** | **p50** | **p75** | **SD** |
| --- | --- | --- | --- | --- | --- | --- |
| **Children** | 136 | 57.09 | 3 | 12 | 46 | 284.20 |
| **Adults** | 41 | 13.68 | 7 | 30 | 53 | 141.73 |

Table D: Scar assessment initial and final (VSS); n(%)

| **Final**  **Initial** | **2** | **3** | **4** | **5** | **6** | | **7** | **8** | **9** | **10 or +** |
| --- | --- | --- | --- | --- | --- | --- | --- | --- | --- | --- |
| **2** | - | - | - | 1 (50.0) | - | | - | - | - | - |
| **3** | - | - | - | - | 1 (50.0) | | 1 (50.0) | - | - | - |
| **4** | 1 (11.1) | 2 (22.2) | 5 (55.6) | - | 1 (11.1) | | - | - | - | - |
| **5** | - | 2 (11.1) | 8 (44.4) | 5 (27.8) | 3 (16.7) | | - | - | - | - |
| **6** | - | 4 (12.5) | 6 (18.8) | 11 (34.4) | 7 (21.9) | | - | 4 (12.5) | - | - |
| **7** | 1 (2.8) | 1 (2.8) | 6 (16.7) | 11 (30.6) | 13 (36.1) | | 1 (2.8) | 2 (5.6) | - | 1 (2.8) |
| **8** | - | 1 (2.9) | 2 (5.7) | 4 (11.4) | 11 (31.4) | | 9 (25.7) | 6 (17.1) | 2 (5.7) | - |
| **9** | - | - | 2 (8.7) | 4 (17.4) | 4 (17.4) | | 7 (30.4) | 6 (26.1) | - | - |
| **10 or +** | - | 1 (10.0) | - | - | 1 (10.0) | | 1 (10.0) | 5 (50.0) | - | 2 (20.0) |
| **Average initial (SD)****: 7.1 (1.8)** | | | | | | **Average final (SD): 5.7 (1.7)** | | | | |

Table E: Pain Scores from initial to final (VAS); n (%)

| **Final**  **Initial** | **0** | **1-3** | **4-6** | **7-10** |
| --- | --- | --- | --- | --- |
| **0** | 6 (85.7) | 1 (14.3) | - | - |
| **1-3** | 21 (67.7) | 8 (25.8) | 2 (6.5) | - |
| **4-6** | 33 (46.5) | 27 (38.0) | 11 (15.5) | - |
| **7-10** | 26 (44.8) | 21 (36.2) | 9 (15.5) | 2 (3.5) |
| **Average initial (SD): 5.3 (2.5)** | | | **Average final: 1.4 (1.8)** | |

Table F: Itching Scores, initial and final; n (%)

| **Final**  **Initial** | **0** | **1** | **2** | **3** | **4** | **5** | **6** | **7** | **8** | **9** | **10** |
| --- | --- | --- | --- | --- | --- | --- | --- | --- | --- | --- | --- |
| **0** | 19 (47.5) | 2 (5.0) | 5 (12.5) | 6 (15.0) | 3 (7.5) | 3 (7.5) | 1 (2.5) | 1 (2.5) | - | - | - |
| **1** | 2 (50.0) | - | - | 1 (25.0) | 1 (25.0) | - | - | - | - | - | - |
| **2** | 4 (40.0) | 2 (20.0) | 2 (20.0) | 1 (10.0) | - | - | 1 (10.0) | - | - | - | - |
| **3** | 4 (16.0) | 3 (12.0) | 9 (36.0) | 3 (12.0) | 5 (20.0) | - | - | - | 1 (4.0) | - | - |
| **4** | 2 (9.1) | 1 (4.6) | 8 (36.4) | 8 (36.4) | - | - | 2 (9.1) | - | 1 (4.6) | - | - |
| **5** | 1 (4.8) | 1 (4.8) | 6 (28.6) | 6 (28.6) | 2 (8.5) | 3 (14.3) | 2 (9.5) | - | - | - | - |
| **6** | 2 (11.1) | - | 4 (22.2) | 5 (27.8) | 2 (16.7) | 1 (8.3) | 1 (5.6) | - | 1 (5.6) | - | - |
| **7** | 1 (8.3) | 2 (16.7) | 1 (8.3) | 2 (16.7) | 1 (8.3) | 2 (16.7) | 1 (8.3) | 1 (8.3) | 1 (8.3) | - | - |
| **8** | 1 (9.1) | - | - | 3 (27.3) | 4 (36.4) | 1 (9.1) | - | 1 (9.1) | 1 (9.1) | - | - |
| **9** | **-** | **-** | **-** | **-** | **-** | 1 (33.3) | 1 (33.3) | 1 (33.3) | - | - | - |
| **10** | **-** | **-** | **-** | **-** | **-** | - | - | - | - | - | 1 (100) |
| **Average Initial: 3.7 (2.7)** | | | | | | **Average Final: 2.7 (2.2)** | | | | | |

Table G: Contracture Scores initial and final (Reducibility Score); n (%)

| **Final**  **Initial** | **0** | **1** | **2** | | **3** | **4** | **NA** |
| --- | --- | --- | --- | --- | --- | --- | --- |
| **0** | 21 (95.5) | - | - | | 1 (4.6) | - | - |
| **1** | 16 (59.3) | 11 (40.7) | - | | - | - | - |
| **2** | 10 (28.6) | 16 (45.7) | 7 (20.0) | | 0 | 1 (2.9) | 1 (2.9) |
| **3** | 12 (30.0) | 15 (37.5) | 11 (27.5) | | 2 (5.0) | - | - |
| **4** | 15 (32.6) | 15 (32.6) | 11 (23.9) | | 3 (6.5) | 1 (2.2) | 1 (2.2) |
| **NA** | - | 1 (50.0) | - | | - | - | 1 (50.0) |
| **Average initial: 2.3 (1.4)** | | | | **Average final: 0.8 (0.9)** | | | |

Table H: Functional Ability Scores initial and final (FAB); n (%)

| **Final**  **Initial** | **High** | **Moderate** | | **Mild** | **Dependence** |
| --- | --- | --- | --- | --- | --- |
| **High dependence** | - | - | | - | 3 (100.0) |
| **Moderate dependence** | - | 1 (5.9) | | - | 16 (94.1) |
| **Mild dependence** | - | - | | - | 20 (100.0) |
| **Dependence** | - | - | | - | 36 (100.0) |
| **Average initial: 25.6 (7.1)** | | | **Average final: 34.6 (2.3)** | | |
